# An Overview of the Importance and Value of Porcine Species in Sialic Acid Research

**DOI:** 10.3390/biology11060903

**Published:** 2022-06-11

**Authors:** Oluwamayowa Joshua Ogun, Georg Thaller, Doreen Becker

**Affiliations:** 1 Institute of Animal Breeding and Husbandry, University of Kiel, Olshausenstraße 40, 24098 Kiel, Germany; gthaller@tierzucht.uni-kiel.de; 2 Institute of Genome Biology, Research Institute for Farm Animal Biology (FBN), Wilhelm-Stahl-Allee 2, 18196 Dummerstorf, Germany

**Keywords:** *CMAH*, pig, Neu5Gc, Neu5Ac, sialic acid, red meat

## Abstract

**Simple Summary:**

Humans frequently interact with pigs and porcine meat is the most consumed red meat in the world. In addition, due to the many physiological and anatomical similarities shared between pigs and humans, in contrast to most mammalian species, pigs are a suitable model organism and pig organs can be used for xenotransplantation. However, one major challenge of porcine meat consumption and xenotransplantation is the xenoreactivity between red meat Neu5Gc sialic acid and human anti-Neu5Gc antibodies, which are associated with certain diseases and disorders. Furthermore, pigs express both α2-3 and α2-6 Sia linkages that could serve as viable receptors for viral infections, reassortments, and cross-species transmission of viruses. Therefore, pigs play a significant role in sialic acid research and, in general, in human health.

**Abstract:**

Humans frequently interact with pigs, whose meat is also one of the primary sources of animal protein. They are one of the main species at the center of sialic acid (Sia) research. Sias are sugars at terminals of glycoconjugates, are expressed at the cell surfaces of mammals, and are important in cellular interactions. N-glycolylneuraminic acid (Neu5Gc) and N-acetylneuraminic acid (Neu5Ac) are notable Sias in mammals. Cytidine monophospho-N-acetylneuraminic acid hydroxylase (*CMAH*) encodes the *CMAH* enzyme that biosynthesizes Neu5Gc. Although humans cannot endogenously synthesize Neu5Gc due to the inactivation of this gene by a mutation, Neu5Gc can be metabolically incorporated into human tissues from red meat consumption. Interactions between Neu5Gc and human anti-Neu5Gc antibodies have been associated with certain diseases and disorders. In this review, we summarized the sialic acid metabolic pathway, its regulation and link to viral infections, as well as the importance of the pig as a model organism in Sia research, making it a possible source of Neu5Gc antigens affecting human health. Future research in solving the structures of crucial enzymes involved in Sia metabolism, as well as their regulation and interactions with other enzymes, especially *CMAH*, could help to understand their function and reduce the amount of Neu5Gc.

## 1. Introduction

Research on sialic acids (Sias) is an integral and essential aspect in the field of glycobiology, which is the study of the structure, biosynthesis, and biology of glycans (sugar chains). Sias are negatively charged monosaccharides expressed on the mammalian cell surface and are essential molecules of life due to their role in different cellular processes [1,2]. Sias are made of 9-carbon backbone acidic sugars and are found at the terminals of glycan chains of glycoconjugates, such as glycoproteins and glycolipids of vertebrate cell surfaces [3]. Sias play a crucial role in stabilizing membranes due to their terminal position. Consequently, Sias are involved in cell–cell interaction and cell–microenvironment interaction (comprehensive details in *Essentials of Glycobiology* (2nd edition) by Varki and Schauer [2]). They regulate receptor binding by controlling transmembrane signaling, fertilization, and cell differentiation. Additionally, Sias are one of the primary molecules responsible for conveying inhibitory signals during the innate immune response, e.g., the linkage between sialylated glycans and Sia receptors on tumor cells can mediate the evasion of immune surveillance [4]. Sias also serve as the primary contact between pathogens and host cells because of their terminal position at the cell surface. For example, viral proteins, hemagglutinin (HA), and neuraminidase (NA) interact with the Sias of host cells, making them critical receptors for cell infections [5,6]. Sias can also serve as cell protectors against protease or glycosidases [5,6,7,8].

**Figure 1:**
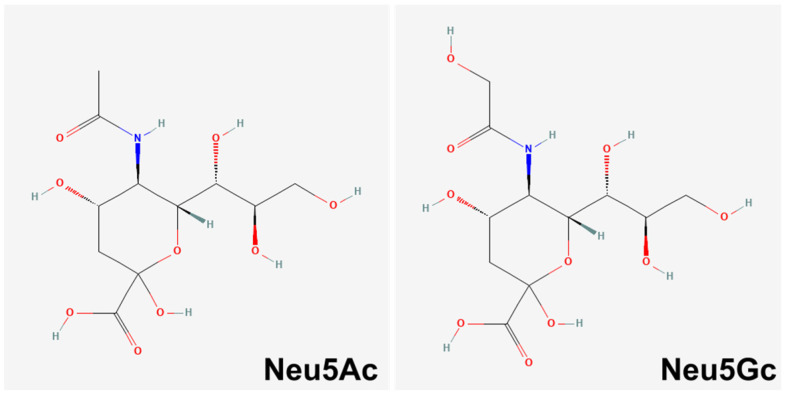
Chemical structure of Neu5Ac and Neu5Gc. Neu5Gc differs by a single oxygen atom that is added by the *CMAH* enzyme. Retrieved from PubChem [18] (Neu5Ac–CID 439197; Neu5Gc–CID 440001).

One of the central focus in sialic acid research is the role of an essential sialic acid sugar molecule known as N-glycolylneuraminic acid (Neu5Gc). Neu5Gc and N-acetylneuraminic acid (Neu5Ac) are the most common Sias in mammals (Figure 1) [9,10,11]. Neu5Gc is biosynthesized by the enzyme cytidine monophospho-N-acetylneuraminic acid hydroxylase (*CMAH*), which is encoded by the *CMAH* gene [9,10].

Neu5Gc is absent in poultry meat and fish, but high contents are found in red meats (beef, pork, and lamb) and dairy products [12]. Although Neu5Gc is not endogenously synthesized in humans due to the inactivation of the *CMAH* gene by a mutation [13], it can be metabolically incorporated into human tissues from red meat and dairy products [12,14] (for detailed information see Varki (2010) [3]). Interactions between the dietary-incorporated Neu5Gc and the circulating human anti-Neu5Gc antibodies have been associated with red meat diet-induced diseases, such as atherosclerosis, type 2 diabetes, and carcinogenesis [15].

Since the domestication of porcine species [16] and probably even before that (i.e., hunting), porcine meat has been a significant source of animal protein. According to the Organization for Economic Co-operation and Development and the United Nation’s Food and Agriculture Organization (OECD-FAO) agricultural report in 2021, porcine meat is the most consumed red meat in the world [17], despite the association of consumption to different diseases. Additionally, pigs are indispensable in biomedical research as model organisms and are also widely used for xenotransplantation [16]. Additionally, porcine sialic acid plays a key role in viral cross-species transmission and xenosialitis, which could be intensified by red meat consumption.

## 2. The Sialic Acid Metabolic Pathway

**Figure 2:**
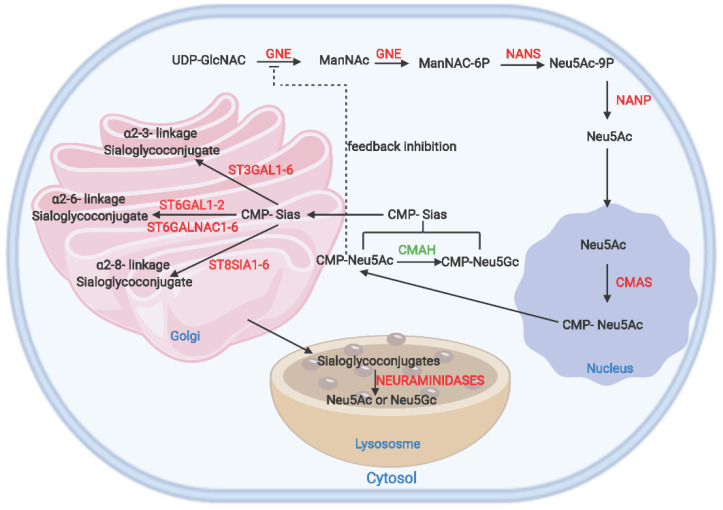
The sialic acid metabolic pathway. The schematic shows the different enzymes involved and each step’s respective localization. Feedback inhibition by CMP-Neu5Ac on GNE is also shown. The *CMAH* that biosynthesizes Neu5Gc is highlighted in green (*CMAH*). The pathway is modified from the REACTOME sialic metabolic pathway [25]. Created with BioRender.com (accessed on 9 March 2022).

Sias belong to the α-keto acid family with more than 50 sialic forms in nature. The most common Sias in mammals are Neu5Ac and the non-human Neu5Gc [9,10,11]. A detailed representation of the sialic acid metabolic pathway is shown in Figure 2. The initiation of sialic acid biosynthesis in mammalian cells occurs in the cytosol [11]. It is catalyzed by a bifunctional enzyme, a key regulator of sialic acid metabolism, called the uridinediphosphate-N-acetylglucosamine 2-epimerase/N-acetylmannosamine kinase (GNE/MNK or simply GNE) [19,20]. The GNE enzyme first catalyzes the conversion of UDP-N-acetylglucosamine (UDP-GlcNAc) to N-acetylmannosamine (ManNAc). This important starting compound also serves as a precursor in many glycan metabolic pathways [19]. The second step involves the phosphorylation of ManNAc to ManNAc-6-phosphate (ManNAc-6-P) by GNE [20].

The following metabolic step is catalyzed by the Neu5Ac-9-phosphate synthase (NANS) which condenses phosphoenolpyruvate with ManNAc-6-P to produce Neu5Ac-9-P [10]. Neu5Ac-9-phosphate phosphatase (NANP) dephosphorylates Neu5Ac-9-P to Neu5Ac [2]. Then, Neu5Ac is transferred into the nucleus from the cytosol, where cytidine monophosphate-Sia synthase (CMAS) transfers cytidine monophosphate (CMP) residue from cytidine triphosphate (CTP) to Neu5Ac to form CMP-Neu5Ac [21]. This activated CMP-Neu5Ac is transported back to the cytosol [22]. In the cytosol, an additional metabolic step takes place, where the CMP-Neu5Ac is converted to CMP-Neu5Gc by *CMAH* [23]. This particular step occurs in porcine species and other mammals, but is absent in humans due to *CMAH* inactivation caused by a 92 bp deletion that occurred probably 2–3 million years ago [13,24]. SLC35A1, a CMP sialic acid carrier, transports CMP-Neu5Gc [11] and CMP-Neu5Ac [25] to the Golgi, where it forms glycoconjugates by transferring Sia residues from its activated sugar cytidine 5′-monophosphate sialic acid (CMP-Sia) to various glycoconjugate terminals [26] catalyzed by different sialyltransferases (STs) [10].

Wickramasinghe and Medrano [11] reviewed a detailed description of the enzymatic functions of the different STs. The STs are divided into four groups based on their Sia linkages and acceptor specificity: ST3GAL, ST6GAL, ST6GALNAC, and ST8SIA. Neuraminidases play a vital role in regulating sialic acid degradation by removing glycosidic links of Sia residues. Neuraminidase 3 (NEU3) located in the plasma membrane is a critical regulator in transmembrane signaling and hydrolyses Neu5Ac from glycoconjugates. Neuraminidase 1 (NEU1) located in lysosomes and neuraminidase 4 (NEU4) also hydrolyze Sia residues from glycoconjugates, but in contrast to NEU1 and NEU3, NEU4 is located in lysosomes, mitochondria, and the endoplasmic reticulum. In the cytosol, neuraminidase 2 (NEU2) breaks down α2-3 sialylated glycoconjugates. The sialin (SLC17A5) enzyme is responsible for the co-transportation of free Sia from the lysosomal lumen to the cytosol, where it can be reused or degraded. In a final step, Neu5Ac and Neu5Gc are cleaved to pyruvate and ManNAc and ManGc, respectively, by N-acetylneuraminate pyruvate lyase (NPL).

## 3. Porcine *CMAH* Structure

*CMAH* catalyzes the conversion of Neu5Ac to Neu5Gc. While this process is predominant in most mammals, Neu5Gc is not endogenously synthesized in humans [23]. The absence of Neu5Gc in humans is presumably associated with improved brain functions [24] and positive influences on the immune system [27]. However, Neu5Gc is metabolically incorporated into human tissues from red meat and dairy products. The interaction between the incorporated Neu5Gc and the circulating human anti-Neu5Gc antibodies [28,29] has been associated with type-2 diabetes, carcinogenesis, and atherosclerosis [14]. The *CMAH* gene is located on chromosome seven (SSC7) of the porcine genome. Porcine *CMAH* (*pCMAH*) consists of 13 exons with the total annotated spliced exon length of 1734 bp (NM_001113015.1) encoding a protein with 577 amino acids (NP_001106486.1). Song et al. [30] reported an additional exon (exon 14). Results obtained with the basic local alignment search tool for nucleotide (BLASTn) show sequence homology of 92.62% and 92.20% with *CMAH* genes of other red meat species, such as *Bos taurus* (XM_024984024) and *Ovis aries* (XM_027958397), respectively.

To better understand the *pCMAH* enzyme, knowledge of the 3D structure with its binding sites is necessary; however, this remains a significant challenge in *pCMAH* research. So far, a search in protein databases did not result in any matches of homologous sequences with close identity. InterProscan has proven to be one of the most effective tools to predict important binding sites and protein domains [31]. It gives a detailed functional analysis of proteins by classifying them into families and predicting domains and important sites using different predictive models provided by 14 different databases that make up the InterProscan consortium [31]. The InterProscan prediction shows that *CMAH* contains a Rieske domain between amino acid positions 6–112, and this supports the assumption that the activity of *CMAH* is dependent on the cytochrome b5 system [32]. *CMAH* also possesses an iron-containing, Rieske-type prosthetic group. Its activity depends on NAD(P)H (dihydronicotinamide adenine dinucleotide phosphate), which requires electron transport proteins cytochrome b5 reductase and cytochrome b5 to be active [32]. Additionally, the protein sequence has significant motifs for [2Fe-2S] cluster-binding sites at amino acid positions 54, 56, 57, 75, 78, and 80 [31].

Song et al. showed that *pCMAH* mRNA expression is tissue- and organ-specific [30]. It was observed that *pCMAH* is highly expressed in the small intestine and spleen, and moderately expressed in the rectum, tongue, testes, liver, and colon. The study identified two alternative spliced forms of the 5′UTR (untranslated region), namely 5′UTR-1 and 5′UTR-2, having different expression patterns in various pig tissues, except for the rectum. 5′UTR-1 was mainly expressed in the small intestine and colon. However, 5′UTR-2 was the dominant form in the spleen, tongue, testicle, kidney, and liver. In all mammals, the *CMAH* gene is downregulated in the brain, which also had the lowest Neu5Gc amount compared to other tissues of the body [33], but during the developmental stage, porcine skeletal muscle contained the lowest concentration of Neu5Gc compared to other tissues [34]. Lepers et al. [35] showed that *CMAH* is the primary factor determining the level of Neu5Gc in cells. The study showed a correlation between enzyme activity and Neu5Gc concentration, suggesting that the animal’s metabolic state might also affect the Neu5Gc concentration. The mRNA level in developing pig jejunum correlated with variations in the enzyme activity, implying that the formation of Neu5Gc is regulated by the transcription of the *CMAH* gene [36].

**Figure 3:**
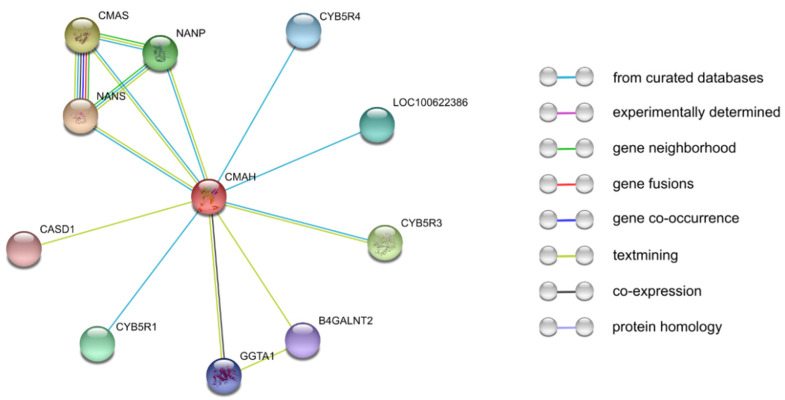
*CMAH* protein–protein interaction network determined by STRING analysis. The interaction sources comprise curated databases, experimental determined, gene neighborhood, gene fusions, gene co-occurrence, text mining, co-expression, and protein homology. The STRING database predicted 10 functional partners [37].

Another critical activity that generally regulates the functions of enzymes is the protein–protein interaction network. In addition to the unresolved 3D structure of *pCMAH*, another main challenge yet to be resolved is the enzyme’s interactions with other proteins. We used a popular protein–protein interaction prediction tool (STRING), which is an efficient database that computationally predicts protein–protein associations by integrating all publicly available protein–protein interaction information sources and covers up to 14,094 different organisms. Additionally, STRING has essential features to perform gene-set enrichment analysis using popular classification systems, such as Gene Ontology and the Kyoto Encyclopedia of Genes and Genomes (KEGG) [37]. The interaction prediction showed that *pCMAH* probably interacts with 10 different proteins (Figure 3). *CMAH* interaction with CMAS, NANP, and NANS might be a result of their direct involvement in sialic acid metabolism. The predicted interaction between *CMAH* and the cytochrome b5 proteins (CYB5R1, CYB5R3, CYB5R4, and LOC100622386) could be a result of *CMAH* dependence on NAD(P)H cofactor and electron transport proteins (cytochrome b5 reductase and cytochrome b5) for *CMAH* activity (Kozutsumi et al., 1990). β-1,4-N-acetyl-galactosaminyl transferase 2 (B4GALNT2), gamma-glutamyltransferase 1 (GGTA1), and *CMAH* are categorized as xenoreactive antigen proteins [38]; therefore, their interaction prediction could just be a result of their co-occurrence in the scientific literature. The interaction between N-acetylneuraminate 9-O-acetyltransferase (CASD1) and *CMAH* could be a result of the role of CASD1 in the modification of sialic acids [39]. Nevertheless, the STRING prediction might serve as a basis for future research in understanding *CMAH* protein–protein interactions.

## 4. Pigs as a Supply for Food Protein

**Figure 4:**
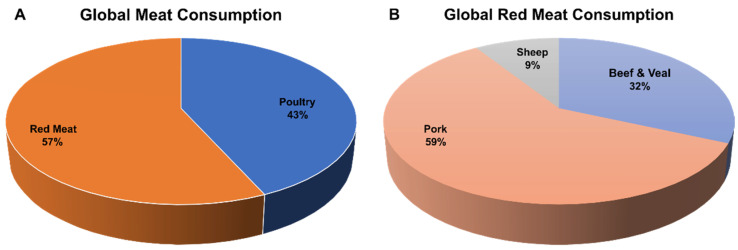
Global meat and red meat consumption. Percentages of (**A**) global meat and (**B**) red meat consumption in 2021, according to the OECD-FAO agricultural report of 2022 [17].

One of the main drivers for the domestication of pigs was the supply of meat as a food protein. The majority of today’s breeds emanated from the breed development’s contribution due to European and Chinese breeding efforts [40,41,42]. In recent years, there has been an increase in the dietary transition towards animal protein, driven by rising population growth [43,44], resulting also in an increasing demand for pig products. According to the Organization for Economic Co-operation and Development and the United Nation’s Food and Agriculture Organization (OECD-FAO) agricultural report in 2021, the top ten pork-meat-consuming nations are Korea, Vietnam, Chile, United States, China, OECD (includes all European countries, excluding Iceland), Switzerland, Paraguay, Norway, and Russia with 31.6, 25.9, 25.0, 23.9, 23.7, 22.8, 22.4, 21.6, 21.1, and 20.9 kg/capita, respectively [17]. Economically, the meat industry also provides a source of livelihood for many people. Poultry meat and red meat consumption account for approximately 43% and 57% of the global meat consumption, respectively (Figure 4A). In terms of red meat consumption, pork meat accounts for the highest proportion with 59%, while beef (veal included) and sheep account for 32% and 9%, respectively (Figure 4B).

Red meat contains high biological value protein with micronutrients essential for healthy living. Red meat comprises essential omega-3 polyunsaturated fats, niacin, vitamin B6, vitamin B12, phosphorus, zinc, and iron. It provides the body with all essential amino acids and a higher concentration of vitamins compared to other protein sources [45,46]. In addition, red meat serves as a significant source of choline, an important compound that serves as a precursor to many molecules, such as neurotransmitters and membrane phospholipids [45,46]. Despite all the nutritional value, research studies have continuously associated red meat with different diseases and disorders [47,48,49,50,51]. This is mainly due to the metabolic incorporation of Neu5Gc into human tissues from red meat. The daily consumption of red meat products containing Neu5Gc can be metabolized by human cells and incorporated into glycoconjugates, and might be a potential xeno-autoantigen in humans [52], which has been linked to an increased risk of cardiovascular disease and carcinomas [3]. Ji et al. [34] looked at Sia concentrations in different porcine organs and found that the Neu5Gc concentration varies between 21.5% (kidney) and 81.5% (spleen). A study by Samraj et al. [14] provides a summary of the Neu5Gc concentration in various diets. Poultry (hen egg, turkey, and chicken) and dairy products, such as butter containing no Neu5Gc; however, whole milk, cow’s milk cheeses, and goat’s milk cheeses contain 2, 10–22, and 43 µg Neu5Gc per g, respectively. In addition, lamb, beef, and pork contain 14, 25–231, and 7–40 µg Neu5Gc per g, respectively [14].

## 5. Pigs as a Model Organism

Commonly, mice are used as a model organism due to their body size, life cycle span, reproduction time, and availability. In comparison to pigs, mice lack basic features of human disorders [53]. Pigs share a high number of physiological and anatomical characteristics with humans [16,54,55]. These shared features make pigs a suitable model organism for surgical, histological, and pathophysiological studies; therefore, in clinical studies, pigs have replaced dogs for surgical training [16,55]. In addition, studies on human skin wounds and diseases using pigs as model showed highly concordant results [56]. The pig is also a useful animal model in studies of inherited disorder, such as Schmid metaphyseal chondrodysplasia (SMCD) in humans, which is associated with dwarfism. This is caused by a similar mutation in the *COL10A1* gene [57]. Lastly, pigs are also the best suitable animal model for human atherosclerosis, due to similarities in porcine and human lipoprotein metabolism [55] and human ventricular septal defect [58].

Many challenges posed by the use of large animals as a model organism are due to their body size and cost in maintenance. For pigs, this was addressed by breeding specialized minipigs for research purposes [16,59]. Many minipig strains, which have been bred using crossbreeding programs, exist [16,54,59]. In dermatological and ocular studies, minipigs serve as a suitable replacement for rabbits and guinea pigs [60,61,62]. The advantage of minipigs might not only be that they are mostly outbred strains, but also have other unique characteristics, such as the lack of skin pigmentation, a smaller body size compared to other pigs, and their age at sexual maturity [60].

### Significance for Xenotransplantation

Xenotransplantation involves the use of animal tissues or organs in humans [63]. In recent years, there has been an increase in the use of animal organs as a replacement for failed human organs. In 2017, according to data from the European Committee on Organ transplantation (CDPTO), more than 144,000 patients were recorded on transplant waiting lists. At least six new patients were added every hour across Europe, while just 43,000 patients are receiving a transplant yearly [64]. Shared similarities and features of porcine organs and their metabolism with humans make pigs a promising target for xenotransplantation [65,66].

**Figure 5:**
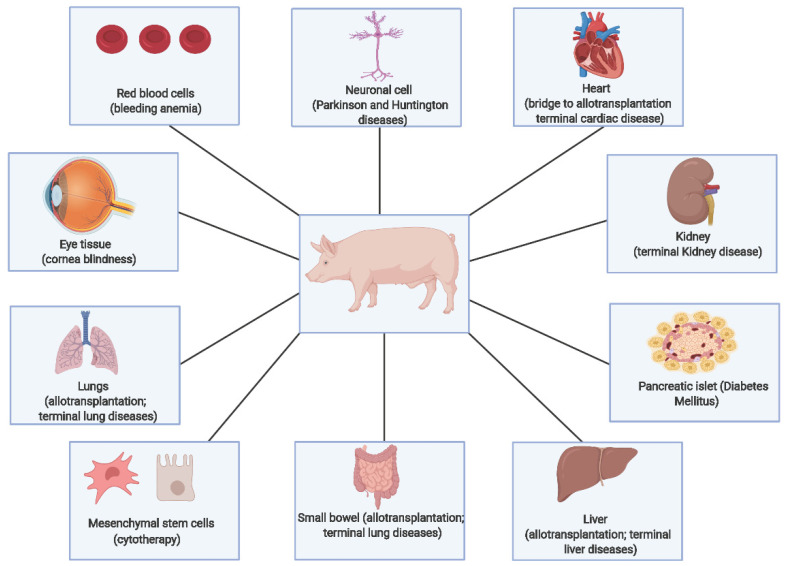
Potential applications of different pig organs and tissues in xenotransplantation. Adapted from Ekser et al. [90]. Created with BioRender.com (accessed on 9 March 2022).

So far, different pig tissues or organs (such as kidney, heart, liver, spleen, bone marrow, skin graft, and hepatocytes) have been transplanted to humans [7]. Figure 5 shows different diseases and disorders for which xenotransplantation could be used as an alternative therapy. Aside from organ transplantation, xenotransplantation also includes transplantation of pig cells (such as neuronal and pancreatic islet cells) and the use of viable pig cells or organs as biomedical devices (such as bioprosthetic heart valves) [7]. Despite the numerous applications of pig tissues and organs in xenotransplantation, the main challenge is graft rejection caused by the interaction of the porcine xenoantigens with human xenoreactive antibodies.

Most of the xenoreactive antibodies in human serum bind to the α-galactose (αGal) glycoprotein epitope [67]. This particular molecule is biosynthesized by the α-1,3-galactosyltransferase enzyme (GGTA1) in pigs [68] encoded by the *GGTA1* gene, which is absent in humans [69,70]. Although the knockout (KO) of *GGTA1* in pigs improved kidney transplant survival in pig to non-human primates, xenoreactivities were still observed [71,72]. According to Chen et al. [73], the KO of other xenoantigen genes is imperative to eliminate graft rejection. A study by Byrne in 2015 indicated that porcine beta-1,4-N-acetyl-galactosaminyltransferase 2, encoded by *B4GALNT2,* also acts as an immunogenic xenotransplantation antigen in pig-to-baboon xenotransplantation [74,75].

In 2010, Song et al. [30] observed that the biosynthesis of Neu5Gc by *pCMAH* could act as a xenoantigen in humans. Additionally, they showed that the increase in xenoantigenicity in pig-transfected cell lines does not depend on the αGal antigen, concluding that Neu5Gc is a significant xenoantigen. Furthermore, after porcine skin grafting for patients that suffered from severe burns, Neu5Gc was the main non-αGal xenoantigen recognized [76]. Unlike αGal antigen, the anti-Neu5Gc effect is not easily degraded in the body or suppressed by immunosuppressive drugs in patients [77]. The continuous exposure to Neu5Gc residues used as biodevices in various medical treatments may elicit inflammation or xenosialitis. Aside from the Neu5Gc antigen that arises directly from the transplant, preformed anti-Neu5Gc circulating antibodies (i.e., as a result of red meat consumption) might also affect graft acceptance [52].

Due to the advancement in genomic research in recent years, tools for precise genome editing using specific nucleases to edit gene sequences (such as zinc-finger nucleases (ZFN); transcription activator-like effector nucleases (TALEN); and clustered, regularly interspaced, short palindromic repeats (CRISPR) with (Cas) nucleases (CRISPR/Cas)) are having a huge impact on science [78,79,80]. With regards to the KO of the *CMAH* gene, the pig is the species of interest [73,75,80,81,82,83,84,85,86]. The work by Phelps et al. [68] was regarded as the first to inactivate the αGal antigen in pig cells. As stated earlier, the KO of *GGTA1* did not eradicate xenoreactivity. Instead, an increase in the expression of Neu5Gc was observed when a heart with αGal KO was transplanted to humans [71,87].

To improve the efficiency of xenotransplantation, most studies combine the KO of *CMAH* with other xenoantigen genes [66,81,82,88]. The KO of *CMAH* in combination with *GGTA1* has been shown to reduce antibody binding of human serum compared to a single KO of *GGTA1* [82,89]. Other studies targeted three xenoantigen genes simultaneously (*GGTA1*, *B4GALNT2*, and *CMAH*) by inactivating these genes with CRISPR/Cas9 [38,89]. It was observed that pig valves showed a reduction in both human IgM and IgG binding, when these three xenoreactive antigen genes were deleted [85].

## 6. Porcine Sialic Acid in Infection, Cross-Species Transmission, and Emergence of Novel Viruses

Due to close interactions between animals and humans, challenges arose concerning zoonotic infections, especially of viral nature. These interactions take place between humans and majorly livestock species. Aside from humans frequently interacting with pigs in regards to meat production, certain minipig and pig breeds are also kept as pets.

There are several types of zoonotic viruses that could potentially be pathogenic. Some of these virus families are Orthomyxoviridae, Coronaviridae, Paramyxoviridae, Caliciviridae, Picornaviridae, Reoviridae, Polyomaviridae, Adenoviridae, and Parvoviridae [6]. Orthomyxoviridae is one of the most common families of negative-sense single-stranded RNA viruses [91], consisting of five genera (influenza virus A, B, and C; Thogotovirus; and Isavirus), which are significant pathogens of humans and animals [92]. Influenza virus A, a pathogenic virus common in multiple species, is further categorized based on the type of antigenicity of their surface glycoproteins: hemagglutinins (HA) and neuraminidases (NA). Both HA and NA proteins recognize the same host cell Sias; while the HA (a lectin) is needed for viral attachment to the host cells to initiate virus infection, NA detaches the virus from the cell surface glycoproteins to facilitate the release of the virus progeny and to promote the viral infections of other cells [93].

The world has witnessed many influenza pandemics. An unforgettable pandemic is that of the Spanish flu in 1918, which killed more than 20 million people [94]. Sias in host cells have always played a significant role as receptors for viral attachment due to their terminal position in glycoconjugates [95,96,97,98,99]. Another vital factor in Sias viral interactions is the type of Sia linkage. The common connection of Sias to another residue is via α2-3 or α2-6 linkage to either galactose (Gal) or N-acetylgalactosamine (GalNAc) and α2-8 linkage. Most influenza viruses preferably bind to α2-3- or α2-6-linked Sias in the form of Neu5Acα2-3/6Gal, Neu5Acα2-3/6GalNAc, and Neu5Acα2-6GlcNAc [6].

While human influenza viruses preferentially bind to α2-6-linked Sias, most of avian and equine influenza viruses prefer α2-3-linked Sias as receptors [5,100,101]. A study in 1998 by Ito et al. [100] first showed that the pig trachea expresses both receptors for α2-3 and α2-6 Sia linkage. In 2010, Nelli et al. [101] also detected the extensive expression of both α2-3-Gal and α2-6-Gal receptors in different pig organs. These studies showed that porcine sialic acids could serve as receptors for viruses from avian or/and human origin, thereby providing a potential link to other mammals and serving as a reassortment vessel for influenza viral cross-species transmission. In addition, pigs express both Sias, i.e., Neu5Gc and Neu5Ac.

Three primary conditions must be met for pandemic influenza to occur. Firstly, a new influenza virus strain must emerge to which the human population has little or no immunity. Secondly, the new strain must be able to replicate efficiently in humans and result in disease, and lastly, the virus must be transmittable from human to human. For a novel or modified virus to emerge, reassortment of viral genes (also known as antigenic shift) or antigenic drift must occur [98,102,103]. On the one hand, antigenic shift involves reassortment between different subtypes, and large RNA sequence changes, creating a novel virus. During this process segmented RNA viruses, such as influenza, can exchange their genome segments during co-infection, resulting in different subtypes of viruses that the human population lacks immunity against [98]. On the other hand, antigenic drift is a result of small accumulations of mutations of the virus RNA, which cause changes in the HA and NA of the virus resulting in a new virus strain [102].

Studies have shown that both human and avian influenza viruses can be naturally transmitted to pigs [101,104,105]. Pigs, as influenza virus mixing vessels, were first proposed by Scholtissek et al. in 1990 [106], and since then, several studies came to a similar conclusion [101,104,105,107]. Previous evidence from the H5N1 outbreak in Hong Kong showed that avian viruses could be directly transmitted from birds to various mammals, including pigs [100,108,109]. H1N1 avian influenza viruses (AIV) have been isolated from pigs in the past and were the first detectable AIVs in European pigs since 1979 [110]. The pig’s role as a mixing vessel was further elucidated, when a novel triple reassortant of H3N2 was detected in the late 1990s with the combination of human seasonal H3N2, avian influenza, and the classical H1N1 swine influenza virus [111]. In the early 2000s, this mixed virus further reassorted with classical H1N1 viruses to produce novel viruses H1N1 and H1N2 [112,113]. In 1993, when Castrucci et al. [114] analyzed the internal protein genes of classic H1N1, avian-like H1N1, and human-like H3N2 viruses in Italian pigs, their results showed that human-like H3N2 strains had the internal protein genes of avian-like H1N1 viruses, suggesting a possible reassortment between the avian and human-like viruses. Furthermore, other studies also detected the presence of influenza viruses of avian origin in North American and Asian pigs [112,115].

**Figure 6:**
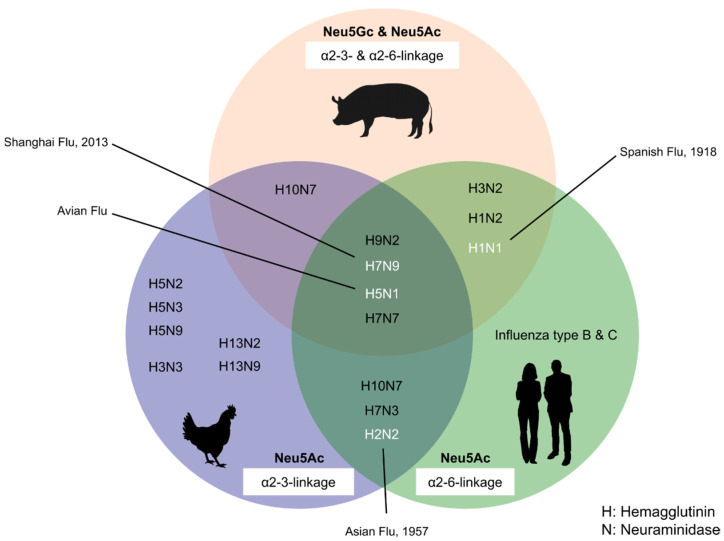
Pigs act as a “mixing vessel” between avian species and humans. Pig possesses both α2-3 and α2-6 linkage and also the two main Sia structures (Neu5Gc and Neu5Ac). Pigs act as a “mixing vessel” between avian species and humans for influenza viruses. Gene swapping can occur in pigs between avian, swine, and human viruses, which can result in the evolution of a novel virus that could lead to pandemics in humans.

Evidence of reassortments of viruses taking place in pigs was also established in Argentina in 2011, when a study showed that two independent reassortments occurred between the H1N1 pandemic influenza virus (H1N1pdm) and the human-like swine influenza virus [116]. In 2015, two novel reassortant viruses of human-like H3N2 and H3N1 influenza A viruses were identified in pigs in the USA [105]. The study showed that HA genes were similar to those of human seasonal H3 strains and closely related to the 2009 H1N1 pandemic virus. While the NA of H3N2 showed similarities with human N2 lineage, the H3N1 was of classical porcine N1 origin [105]. In 2016, in Tianjin, China, novel triple-reassortant H1N1 swine influenza viruses were also detected in pigs. The study’s phylogenetic analysis showed that these novel viruses contained viral proteins from the 2009 pandemic H1N1 and the viral proteins from Eurasian swine and triple-reassortant swine lineages [117]. Figure 6 shows how pigs act as a “mixing vessel” between avian species and humans for influenza viruses.

Furthermore, a study from 2018 looked at H1N1 and H3N2 influenza A viruses of swine origin from pigs, which were isolated between 2013 and 2015 [118]. The study showed that the viruses contained proteins from the Eurasian avian-like (EA) or H1N1 seasonal human-like virus (H3N2), and those from the H1N1/2009 pandemic or EA H1N1, viral proteins from classical swine, and some components from the H1N1 pandemic virus in 2009. The study identified a similar genetic factor that could result in significant transmission and stability in the human population, thereby posing a potential danger for pandemics [118]. Although pigs commonly transmit the influenza virus to humans, the stability within the human population is rare, even though a case of death in a human was reported in 1993 in the USA [119]. Considering all this information, the susceptibility of pigs to different influenza viral infections, and the fact that pigs express both Sia linkages and types, pigs serve as an agent for cross-species transmission of novel influenza viruses.

In addition, studies have shown that porcine Sias could act as an attachment receptor for coronaviruses and could also present varieties of Sia linkages or structures as receptor bindings for coronaviruses from different species [97,120,121].

At present, the world is experiencing a pandemic caused by the novel severe acute respiratory syndrome coronavirus 2 (SARS-CoV2). This novel SARS-CoV2 belongs to the family of Coronaviridae, families of positive-sense single-stranded RNA viruses. Four genera make up this family: Alphacoronavirus, Betacoronavirus, Deltacoronavirus, and Gammacoronavirus [91]. While the Deltacoronavirus can infect both mammals and birds, the Alphacoronavirus and Betacoronavirus are peculiar to mammals and Gammacoronaviruses to birds [122]. A unique attribute of this family is that they possess larger genomes compared to other single-stranded RNA viruses, which makes them susceptible to mutations and recombination events that can result in different variants or novel strains of these viruses [123,124].

Opriessnig and Huang [125] reviewed the potential role of pigs in this current pandemic. They discussed the susceptibility of pigs to coronaviruses from different genera. They also stated that, while the human coronavirus targets the respiratory tracts just like SARS-CoV2, intestines are the main targets of the most porcine coronaviruses [125].

Numerous studies have investigated the possibility of cross-species transmission of coronaviruses [123,124,126,127,128]. In 2005, a study by Chen et al. [126] analyzed the cross-species transmission of SARS-associated coronavirus from humans to pigs and showed that human SARS-CoV can infect pigs. Furthermore, sequence analysis carried out by Woo et al. [128] showed that porcine deltacoronavirus (PDCoV) could have emanated from a recombination between sparrow coronavirus and bulbul coronavirus. PDCoV can also infect other species, including calves, chicken, turkeys, mice [129,130,131,132], and humans [133]. Unlike the evidence of cross-species transmission of SARS-CoV between humans and pigs, as of the time of writing this review, there is no clear evidence of transmission of the current novel SARS-CoV2 between humans and pigs. However, due to the susceptibility of coronaviruses to mutations and recombination events, as well as the fact that pigs have both Sia linkages and structures, it should not be neglected that pigs serve as a potential agent for cross-species transmission of novel coronaviruses.

## 7. Conclusions

Pigs are among the common mammalian species with direct and frequent interactions with humans, aside from being one of the primary sources of animal protein. The role of pigs as model organisms, their significance for xenotransplantation coupled with their roles in cross-species transmission and the possibility of the emergence of novel influenza viruses and coronaviruses, as well as their uniqueness in terms of possessing both α2-3 and α2-6 Sia linkages and the two main Sia structures, Neu5Gc and Neu5Ac, give them an important and fundamental role in sialic acid research and, in general, in human health. Although, over the years, numerous studies have been carried out with different approaches concerning Sia linkages and structures, the three-dimensional (3D) structural information of the enzymes directly involved in the synthesis of Sias is still unknown. The 3D structures can be resolved using different approaches, such as X-ray crystallography, nuclear magnetic resonance, and cryo-electron microscopy. This information could provide a better understanding of the domains, motifs, and folds in protein structure. Furthermore, details on binding sites, ligands, and protein interactions, as well as the impact of mutations and druggability, could be studied. Hence, porcine meat and organs with reduced or even absence of Neu5Gc might become available in the future.

## Data Availability

Not applicable.

## References

[1] Varki A. (2008). Sialic acids in human health and disease. Trends Mol. Med..

[2] Varki A., Schauer R., Varki A., Cummings R.D., Esko J.D., Freeze H.H., Stanley P., Bertozzi C.R., Hart G.W., Etzler M.E. (2009). Sialic Acids. Essentials of Glycobiology.

[3] Varki A. (2010). Colloquium paper: Uniquely human evolution of sialic acid genetics and biology. Proc. Natl. Acad. Sci. USA.

[4] Zhou X., Yang G., Guan F. (2020). Biological Functions and Analytical Strategies of Sialic Acids in Tumor. Cells.

[5] Matrosovich M., Herrler G., Klenk H.D. (2015). Sialic Acid Receptors of Viruses. Top. Curr. Chem..

[6] Suzuki Y., Ito T., Suzuki T., Holland R.E., Chambers T.M., Kiso M., Ishida H., Kawaoka Y. (2000). Sialic acid species as a determinant of the host range of influenza A viruses. J. Virol..

[7] Magre S., Takeuchi Y., Bartosch B. (2003). Xenotransplantation and pig endogenous retroviruses. Rev. Med. Virol..

[8] Payne S., Payne S. (2017). Family Coronaviridae. Viruses: From Understanding to Investigation.

[9] Angata T., Varki A. (2002). Chemical diversity in the sialic acids and related alpha-keto acids: An evolutionary perspective. Chem. Rev..

[10] Kooner A.S., Yu H., Chen X. (2019). Synthesis of N-Glycolylneuraminic Acid (Neu5Gc) and Its Glycosides. Front. Immunol..

[11] Wickramasinghe S., Medrano J.F. (2011). Primer on genes encoding enzymes in sialic acid metabolism in mammals. Biochimie.

[12] Samraj A.N., Laubli H., Varki N., Varki A. (2014). Involvement of a non-human sialic Acid in human cancer. Front. Oncol..

[13] Chou H.H., Takematsu H., Diaz S., Iber J., Nickerson E., Wright K.L., Muchmore E.A., Nelson D.L., Warren S.T., Varki A. (1998). A mutation in human CMP-sialic acid hydroxylase occurred after the Homo-Pan divergence. Proc. Natl. Acad. Sci. USA.

[14] Samraj A.N., Pearce O.M., Laubli H., Crittenden A.N., Bergfeld A.K., Banda K., Gregg C.J., Bingman A.E., Secrest P., Diaz S.L. (2015). A red meat-derived glycan promotes inflammation and cancer progression. Proc. Natl. Acad. Sci. USA.

[15] Dhar C., Sasmal A., Varki A. (2019). From ”Serum Sickness” to “Xenosialitis”: Past, Present, and Future Significance of the Non-human Sialic Acid Neu5Gc. Front. Immunol..

[16] Gutierrez K., Dicks N., Glanzner W.G., Agellon L.B., Bordignon V. (2015). Efficacy of the porcine species in biomedical research. Front. Genet..

[17] OECD Agricultural Output—Meat Consumption. http://data.oecd.org/agroutput/meat-consumption.htm.

[18] Kim S., Chen J., Cheng T., Gindulyte A., He J., He S., Li Q., Shoemaker B.A., Thiessen P.A., Yu B. (2021). PubChem in 2021: New data content and improved web interfaces. Nucleic Acids Res..

[19] Hinderlich S., Weidemann W., Yardeni T., Horstkorte R., Huizing M. (2015). UDP-GlcNAc 2-Epimerase/ManNAc Kinase (GNE): A Master Regulator of Sialic Acid Synthesis. Top. Curr. Chem..

[20] Kurochkina N., Yardeni T., Huizing M. (2010). Molecular modeling of the bifunctional enzyme UDP-GlcNAc 2-epimerase/ManNAc kinase and predictions of structural effects of mutations associated with HIBM and sialuria. Glycobiology.

[21] Traving C., Schauer R. (1998). Structure, function and metabolism of sialic acids. Cell. Mol. Life Sci..

[22] Huizing M. (2005). Disease mechanisms associated with mutations of the GNE gene. Drug Discov. Today Dis. Mech..

[23] Varki A. (2001). Loss of N-glycolylneuraminic acid in humans: Mechanisms, consequences, and implications for hominid evolution. Yearb. Phys. Anthr..

[24] Chou H.H., Hayakawa T., Diaz S., Krings M., Indriati E., Leakey M., Paabo S., Satta Y., Takahata N., Varki A. (2002). Inactivation of CMP-N-acetylneuraminic acid hydroxylase occurred prior to brain expansion during human evolution. Proc. Natl. Acad. Sci. USA.

[25] Jassal B., Matthews L., Viteri G., Gong C.Q., Lorente P., Fabregat A., Sidiropoulos K., Cook J., Gillespie M., Haw R. (2020). The reactome pathway knowledgebase. Nucleic Acids Res..

[26] Chen X., Varki A. (2010). Advances in the Biology and Chemistry of Sialic Acids. ACS Chem. Biol..

[27] Alisson-Silva F., Liu J.Z., Diaz S.L., Deng L.Q., Gareau M.G., Marchelletta R., Chen X., Nizet V., Varki N., Barrett K.E. (2018). Human evolutionary loss of epithelial Neu5Gc expression and species-specific susceptibility to cholera. PLoS Pathog..

[28] Malykh Y.N., Schauer R., Shaw L. (2001). N-Glycolylneuraminic acid in human tumours. Biochimie.

[29] Tangvoranuntakul P., Gagneux P., Diaz S., Bardor M., Varki N., Varki A., Muchmore E. (2003). Human uptake and incorporation of an immunogenic nonhuman dietary sialic acid. Proc. Natl. Acad. Sci. USA.

[30] Song K.H., Kang Y.J., Jin U.H., Park Y.I., Kim S.M., Seong H.H., Hwang S., Yang B.S., Im G.S., Min K.S. (2010). Cloning and functional characterization of pig CMP-N-acetylneuraminic acid hydroxylase for the synthesis of N-glycolylneuraminic acid as the xenoantigenic determinant in pig-human xenotransplantation. Biochem. J..

[31] Mitchell A.L., Attwood T.K., Babbitt P.C., Blum M., Bork P., Bridge A., Brown S.D., Chang H.Y., El-Gebali S., Fraser M.I. (2019). InterPro in 2019: Improving coverage, classification and access to protein sequence annotations. Nucleic Acids Res..

[32] Kozutsumi Y., Kawano T., Yamakawa T., Suzuki A. (1990). Participation of Cytochrome-B5 in Cmp-N-Acetylneuraminic Acid Hydroxylation in Mouse-Liver Cytosol. J. Biochem..

[33] Kawano T., Koyama S., Takematsu H., Kozutsumi Y., Kawasaki H., Kawashima S., Kawasaki T., Suzuki A. (1995). Molecular-Cloning of Cytidine Monophospho-N-Acetylneuraminic Acid Hydroxylase—Regulation of Species-Specific and Tissue-Specific Expression of N-Glycolylneuraminic Acid. J. Biol. Chem..

[34] Ji S., Wang F., Chen Y., Yang C., Zhang P., Zhang X., Troy F.A., Wang B. (2017). Developmental changes in the level of free and conjugated sialic acids, Neu5Ac, Neu5Gc and KDN in different organs of pig: A LC-MS/MS quantitative analyses. Glycoconj. J..

[35] Lepers A., Shaw L., Schneckenburger P., Cacan R., Verbert A., Schauer R. (1990). A study on the regulation of N-glycoloylneuraminic acid biosynthesis and utilization in rat and mouse liver. Eur. J. Biochem..

[36] Malykh Y.N., King T.P., Logan E., Kelly D., Schauer R., Shaw L. (2003). Regulation of N-glycolylneuraminic acid biosynthesis in developing pig small intestine. Biochem. J..

[37] Szklarczyk D., Gable A.L., Nastou K.C., Lyon D., Kirsch R., Pyysalo S., Doncheva N.T., Legeay M., Fang T., Bork P. (2021). The STRING database in 2021: Customizable protein-protein networks, and functional characterization of user-uploaded gene/measurement sets. Nucleic Acids Res..

[38] Estrada J.L., Martens G., Li P., Adams A., Newell K.A., Ford M.L., Butler J.R., Sidner R., Tector M., Tector J. (2015). Evaluation of human and non-human primate antibody binding to pig cells lacking GGTA1/CMAH/beta4GalNT2 genes. Xenotransplantation.

[39] Baumann A.M.T., Bakkers M.J.G., Buettner F.F.R., Hartmann M., Grove M., Langereis M.A., de Groot R.J., Muhlenhoff M. (2015). 9-O-Acetylation of sialic acids is catalysed by CASD1 via a covalent acetyl-enzyme intermediate. Nat. Commun..

[40] Jones G.F., Rothschild M.F., Ruvinsky A. (1998). Genetic Aspects of Domestication, Common Breeds and Their Origin. The Genetics of the Pig.

[41] Amills M., Clop A., Ramírez O., Pérez-Enciso M. (2010). Origin and Genetic Diversity of Pig Breeds. eLS.

[42] Bosse M., Megens H.J., Frantz L.A., Madsen O., Larson G., Paudel Y., Duijvesteijn N., Harlizius B., Hagemeijer Y., Crooijmans R.P. (2014). Genomic analysis reveals selection for Asian genes in European pigs following human-mediated introgression. Nat. Commun..

[43] Lassaletta L., Billen G., Romero E., Garnier J., Aguilera E. (2014). How changes in diet and trade patterns have shaped the N cycle at the national scale: Spain (1961–2009). Reg. Environ. Change.

[44] Bai Z.H., Ma W.Q., Ma L., Velthof G.L., Wei Z.B., Havlik P., Oenema O., Lee M.R.F., Zhang F.S. (2018). China’s livestock transition: Driving forces, impacts, and consequences. Sci. Adv..

[45] Ahmad R.S., Imran A., Hussain M.B., Arshad M.S. (2018). Nutritional Composition of Meat. Meat Science and Nutrition.

[46] Williams P. (2007). Nutritional composition of red meat. Nutr. Diet.

[47] Sinha R., Chow W.H., Kulldorff M., Denobile J., Butler J., Garcia-Closas M., Weil R., Hoover R.N., Rothman N. (1999). Well-done, grilled red meat increases the risk of colorectal adenomas. Cancer Res..

[48] McAfee A.J., McSorley E.M., Cuskelly G.J., Moss B.W., Wallace J.M.W., Bonham M.P., Fearon A.M. (2010). Red meat consumption: An overview of the risks and benefits. Meat Sci..

[49] Pan A., Sun Q., Bernstein A.M., Schulze M.B., Manson J.E., Stampfer M.J., Willett W.C., Hu F.B. (2012). Red meat consumption and mortality: Results from 2 prospective cohort studies. Arch. Intern. Med..

[50] Bouvard V., Loomis D., Guyton K.Z., Grosse Y., Ghissassi F.E., Benbrahim-Tallaa L., Guha N., Mattock H., Straif K., International Agency for Research on Cancer Monograph Working Group (2015). Carcinogenicity of consumption of red and processed meat. Lancet Oncol..

[51] Wolk A. (2017). Potential health hazards of eating red meat. J. Intern. Med..

[52] Perota A., Galli C. (2019). N-Glycolylneuraminic Acid (Neu5Gc) Null Large Animals by Targeting the CMP-Neu5Gc Hydroxylase (CMAH). Front. Immunol..

[53] Seok J., Warren H.S., Cuenca A.G., Mindrinos M.N., Baker H.V., Xu W., Richards D.R., McDonald-Smith G.P., Gao H., Hennessy L. (2013). Genomic responses in mouse models poorly mimic human inflammatory diseases. Proc. Natl. Acad. Sci. USA.

[54] Panepinto L.M., Tumbleson M.E., Schook L.B. (1996). Miniature Swine Breeds used Worldwide in Research. Advances in Swine in Biomedical Research.

[55] Swindle M.M., Makin A., Herron A.J., Clubb F.J., Frazier K.S. (2012). Swine as Models in Biomedical Research and Toxicology Testing. Vet. Pathol..

[56] Sullivan T.P., Eaglstein W.H., Davis S.C., Mertz P. (2001). The pig as a model for human wound healing. Wound Repair Regen..

[57] Nielsen V.H., Bendixen C., Arnbjerg J., Sorensen C.M., Jensen H.E., Shukri N.M., Thomsen B. (2000). Abnormal growth plate function in pigs carrying a dominant mutation in type X collagen. Mamm. Genome.

[58] Amin Z., Woo R., Danford D.A., Froemming S.E., Reddy V.M., Lof J., Overman D. (2006). Robotically assisted perventricular closure of perimembranous ventricular septal defects: Preliminary results in Yucatan pigs. J. Thorac. Cardiovasc. Surg..

[59] Köhn F., McAnulty P.A., Dayan A.D., Ganderup N.-C., Hastings K.L. (2012). History and Development of Miniature, Micro and Minipigs. The Minipig in Biomedical Research.

[60] Bollen P., Ellegaard L. (1997). The Gottingen minipig in pharmacology and toxicology. Pharm. Toxicol..

[61] Mahl J.A., Vogel B.E., Court M., Kolopp M., Roman D., Nogues V. (2006). The minipig in dermatotoxicology: Methods and challenges. Exp. Toxicol. Pathol..

[62] Shrader S.M., Greentree W.F. (2018). Gottingen Minipigs in Ocular Research. Toxicol. Pathol..

[63] Chapman L.E., Folks T.M., Salomon D.R., Patterson A.P., Eggerman T.E., Noguchi P.D. (1995). Xenotransplantation and xenogeneic infections. N. Engl. J. Med..

[64] EDQM The European Day for Organ Donation and Transplantation [Fact Sheet]. https://www.edqm.eu/sites/default/files/factsheet_organ_tissue_cell_donation_eodd_2018.pdf.

[65] Aristizabal A.M., Caicedo L.A., Martinez J.M., Moreno M., Echeverri G.J. (2017). Clinical xenotransplantation, a closer reality: Literature review. Cir. Esp..

[66] Tector A.J., Mosser M., Tector M., Bach J.M. (2020). The Possible Role of Anti-Neu5Gc as an Obstacle in Xenotransplantation. Front. Immunol..

[67] Galili U. (2016). Natural anti-carbohydrate antibodies contributing to evolutionary survival of primates in viral epidemics?. Glycobiology.

[68] Phelps C.J., Koike C., Vaught T.D., Boone J., Wells K.D., Chen S.H., Ball S., Specht S.M., Polejaeva I.A., Monahan J.A. (2003). Production of alpha 1,3-galactosyltransferase-deficient pigs. Science.

[69] Galili U. (2005). The alpha-gal epitope and the anti-Gal antibody in xenotransplantation and in cancer immunotherapy. Immunol. Cell Biol..

[70] Labarrere C.A., Woods J.R., Hardin J.W., Campana G.L., Ortiz M.A., Jaeger B.R., Reichart B., Bonnin J.M., Currin A., Cosgrove S. (2011). Early prediction of cardiac allograft vasculopathy and heart transplant failure. Am. J. Transpl..

[71] Miwa Y., Kobayashi T., Nagasaka T., Liu D., Yu M., Yokoyama I., Suzuki A., Nakao A. (2004). Are N-glycolylneuraminic acid (Hanganutziu-Deicher) antigens important in pig-to-human xenotransplantation?. Xenotransplantation.

[72] Ezzelarab M., Ayares D., Cooper D.K. (2005). Carbohydrates in xenotransplantation. Immunol. Cell Biol..

[73] Chen G., Qian H., Starz T., Sun H.T., Garcia B., Wang X.M., Wise Y., Liu Y.Q., Xiang Y., Copeman L. (2005). Acute rejection is associated with antibodies to non-Gal antigens in baboons using Gal-knockout pig kidneys. Nat. Med..

[74] Byrne G.W., Du Z., Stalboerger P., Kogelberg H., McGregor C.G. (2014). Cloning and expression of porcine beta1,4 N-acetylgalactosaminyl transferase encoding a new xenoreactive antigen. Xenotransplantation.

[75] Fischer K., Rieblinger B., Hein R., Sfriso R., Zuber J., Fischer A., Klinger B., Liang W., Flisikowski K., Kurome M. (2020). Viable pigs after simultaneous inactivation of porcine MHC class I and three xenoreactive antigen genes GGTA1, CMAH and B4GALNT2. Xenotransplantation.

[76] Scobie L., Padler-Karavani V., Le Bas-Bernardet S., Crossan C., Blaha J., Matouskova M., Hector R.D., Cozzi E., Vanhove B., Charreau B. (2013). Long-Term IgG Response to Porcine Neu5Gc Antigens without Transmission of PERV in Burn Patients Treated with Porcine Skin Xenografts. J. Immunol..

[77] Couvrat-Desvergnes G., Salama A., Le Berre L., Evanno G., Viklicky O., Hruba P., Vesely P., Guerif P., Dejoie T., Rousse J. (2015). Rabbit antithymocyte globulin-induced serum sickness disease and human kidney graft survival. J. Clin. Investig..

[78] Hauschild J., Petersen B., Santiago Y., Queisser A.L., Carnwath J.W., Lucas-Hahn A., Zhang L., Meng X.D., Gregory P.D., Schwinzer R. (2011). Efficient generation of a biallelic knockout in pigs using zinc-finger nucleases. Proc. Natl. Acad. Sci. USA.

[79] Carlson D.F., Tan W.F., Lillico S.G., Stverakova D., Proudfoot C., Christian M., Voytas D.F., Long C.R., Whitelaw C.B.A., Fahrenkrug S.C. (2012). Efficient TALEN-mediated gene knockout in livestock. Proc. Natl. Acad. Sci. USA.

[80] Cong L., Ran F.A., Cox D., Lin S.L., Barretto R., Habib N., Hsu P.D., Wu X.B., Jiang W.Y., Marraffini L.A. (2013). Multiplex Genome Engineering Using CRISPR/Cas Systems. Science.

[81] Burlak C., Bern M., Brito A.E., Isailovic D., Wang Z.Y., Estrada J.L., Li P., Tector A.J. (2013). N-linked glycan profiling of GGTA1/CMAH knockout pigs identifies new potential carbohydrate xenoantigens. Xenotransplantation.

[82] Burlak C., Paris L.L., Lutz A.J., Sidner R.A., Estrada J., Li P., Tector M., Tector A.J. (2014). Reduced binding of human antibodies to cells from GGTA1/CMAH KO pigs. Am. J. Transpl..

[83] Miyagawa S., Matsunari H., Watanabe M., Nakano K., Umeyama K., Sakai R., Takayanagi S., Takeishi T., Fukuda T., Yashima S. (2015). Generation of alpha1,3-galactosyltransferase and cytidine monophospho-N-acetylneuraminic acid hydroxylase gene double-knockout pigs. J. Reprod. Dev..

[84] Martens G.R., Reyes L.M., Li P., Butler J.R., Ladowski J.M., Estrada J.L., Sidner R.A., Eckhoff D.E., Tector M., Tector A.J. (2017). Humoral Reactivity of Renal Transplant-Waitlisted Patients to Cells from GGTA1/CMAH/B4GalNT2, and SLA Class I Knockout Pigs. Transplantation.

[85] Wang R.G., Ruan M., Zhang R.J., Chen L., Li X.X., Fang B., Li C., Ren X.Y., Liu J.Y., Xiong Q. (2018). Antigenicity of tissues and organs from GGTA1/CMAH/beta4GalNT2 triple gene knockout pigs. J. Biomed. Res..

[86] Wang Z.Y., Burlak C., Estrada J.L., Li P., Tector M.F., Tector A.J. (2014). Erythrocytes from GGTA1/CMAH knockout pigs: Implications for xenotransfusion and testing in non-human primates. Xenotransplantation.

[87] Park J.Y., Park M.R., Bui H.T., Kwon D.N., Kang M.H., Oh M., Han J.W., Cho S.G., Park C., Shim H. (2012). alpha1,3-galactosyltransferase deficiency in germ-free miniature pigs increases N-glycolylneuraminic acids as the xenoantigenic determinant in pig-human xenotransplantation. Cell. Reprogram..

[88] Lutz A.J., Li P., Estrada J.L., Sidner R.A., Chihara R.K., Downey S.M., Burlak C., Wang Z.Y., Reyes L.M., Ivary B. (2013). Double knockout pigs deficient in N-glycolylneuraminic acid and galactose alpha-1,3-galactose reduce the humoral barrier to xenotransplantation. Xenotransplantation.

[89] Li P., Estrada J.L., Burlak C., Montgomery J., Butler J.R., Santos R.M., Wang Z.Y., Paris L.L., Blankenship R.L., Downey S.M. (2015). Efficient generation of genetically distinct pigs in a single pregnancy using multiplexed single-guide RNA and carbohydrate selection. Xenotransplantation.

[90] Ekser B., Ezzelarab M., Hara H., van der Windt D.J., Wijkstrom M., Bottino R., Trucco M., Cooper D.K. (2012). Clinical xenotransplantation: The next medical revolution?. Lancet.

[91] King A.M.Q., Adams M.J., Carstens E.B., Lefkowitz E.J., IUMS (2012). Virology. Part II: The Viruses—The Negative Sense Single Stranded RNA Viruses. Virus Taxonomy: Ninth Report of the International Committee on Taxonomy of Viruses.

[92] Payne S., Payne S. (2017). Family Orthomyxoviridae. Viruses: From Understanding to Investigation.

[93] Xu G., Suzuki T., Maejima Y., Mizoguchi T., Tsuchiya M., Kiso M., Hasegawa A., Suzuki Y. (1995). Sialidase of swine influenza A viruses: Variation of the recognition specificities for sialyl linkages and for the molecular species of sialic acid with the year of isolation. Glycoconj. J..

[94] Laver G., Garman E. (2002). Pandemic influenza: Its origin and control. Microbes Infect..

[95] Schauer R., Wu A.M., Adams L.G. (1988). Sialic Acids as Antigenic Determinants of Complex Carbohydrates. The Molecular Immunology of Complex Carbohydrates.

[96] Kelm S., Schauer R. (1997). Sialic acids in molecular and cellular interactions. Int. Rev. Cytol..

[97] Schwegmann-Wessels C., Herrler G. (2006). Sialic acids as receptor determinants for coronaviruses. Glycoconj. J..

[98] Bouvier N.M., Palese P. (2008). The biology of influenza viruses. Vaccine.

[99] Schauer R., Kelm S., Reuter G., Roggentin P., Shaw L., Rosenberg A. (1995). Biochemsitry and Role of Sialic Acids. Biology of the Sialic Acids.

[100] Ito T., Couceiro J.N., Kelm S., Baum L.G., Krauss S., Castrucci M.R., Donatelli I., Kida H., Paulson J.C., Webster R.G. (1998). Molecular basis for the generation in pigs of influenza A viruses with pandemic potential. J. Virol..

[101] Nelli R.K., Kuchipudi S.V., White G.A., Perez B.B., Dunham S.P., Chang K.C. (2010). Comparative distribution of human and avian type sialic acid influenza receptors in the pig. BMC Vet. Res..

[102] Webster R.G., Domingo E., Webster R., Holland J. (1999). Antigenic Variation in Influenza Viruses. Origin and Evolution of Viruses.

[103] Treanor J. (2004). Influenza vaccine—Outmaneuvering antigenic shift and drift. N. Engl. J. Med..

[104] Brown I., Klenk H.-D., Matrosovich M.N., Stech J. (2008). The Role of Pigs in Interspecies Transmission. Avian Influenza.

[105] Rajao D.S., Gauger P.C., Anderson T.K., Lewis N.S., Abente E.J., Killian M.L., Perez D.R., Sutton T.C., Zhang J., Vincent A.L. (2015). Novel Reassortant Human-Like H3N2 and H3N1 Influenza A Viruses Detected in Pigs Are Virulent and Antigenically Distinct from Swine Viruses Endemic to the United States. J. Virol..

[106] Scholtissek C. (1990). Pigs as ‘Mixing Vessels’ for the Creation of New Pandemic Influenza A Viruses. Med. Princ. Pract..

[107] Ma W., Kahn R.E., Richt J.A. (2008). The pig as a mixing vessel for influenza viruses: Human and veterinary implications. J. Mol. Genet. Med..

[108] Hinshaw V.S., Bean W.J., Webster R.G., Rehg J.E., Fiorelli P., Early G., Geraci J.R., St Aubin D.J. (1984). Are seals frequently infected with avian influenza viruses?. J. Virol..

[109] Guo Y., Wang M., Kawaoka Y., Gorman O., Ito T., Saito T., Webster R.G. (1992). Characterization of a new avian-like influenza A virus from horses in China. Virology.

[110] Pensaert M., Ottis K., Vandeputte J., Kaplan M.M., Bachmann P.A. (1981). Evidence for the Natural Transmission of Influenza-a Virus from Wild Ducks to Swine and Its Potential Importance for Man. Bull. World Health Organ..

[111] Olsen C.W. (2002). The emergence of novel swine influenza viruses in North America. Virus Res..

[112] Karasin A.I., Schutten M.M., Cooper L.A., Smith C.B., Subbarao K., Anderson G.A., Carman S., Olsen C.W. (2000). Genetic characterization of H3N2 influenza viruses isolated from pigs in North America, 1977–1999: Evidence for wholly human and reassortant virus genotypes. Virus Res..

[113] Karasin A.I., Landgraf J., Swenson S., Erickson G., Goyal S., Woodruff M., Scherba G., Anderson G., Olsen C.W. (2002). Genetic characterization of H1N2 influenza A viruses isolated from pigs throughout the United States. J. Clin. Microbiol..

[114] Castrucci M.R., Donatelli I., Sidoli L., Barigazzi G., Kawaoka Y., Webster R.G. (1993). Genetic Reassortment between Avian and Human Influenza-a Viruses in Italian Pigs. Virology.

[115] Ninomiya A., Takada A., Okazaki K., Shortridge K.F., Kida H. (2002). Seroepidemiological evidence of avian H4, H5, and H9 influenza A virus transmission to pigs in southeastern China. Vet. Microbiol..

[116] Pereda A., Rimondi A., Cappuccio J., Sanguinetti R., Angel M., Ye J., Sutton T., Dibarbora M., Olivera V., Craig M.I. (2011). Evidence of reassortment of pandemic H1N1 influenza virus in swine in Argentina: Are we facing the expansion of potential epicenters of influenza emergence?. Influenza Other Respir. Viruses.

[117] Sun Y.F., Wang X.H., Li X.L., Zhang L., Li H.H., Lu C., Yang C.L., Feng J., Han W., Ren W.K. (2016). Novel triple-reassortant H1N1 swine influenza viruses in pigs in Tianjin, Northern China. Vet. Microbiol..

[118] He P., Wang G., Mo Y., Yu Q., Xiao X., Yang W., Zhao W., Guo X., Chen Q., He J. (2018). Novel triple-reassortant influenza viruses in pigs, Guangxi, China. Emerg. Microbes Infect..

[119] Brown I.H. (2001). The pig as an intermediate host for influenza A viruses between birds and humans. Int. Congr. Ser..

[120] Cui T., Theuns S., Xie J., Van den Broeck W., Nauwynck H.J. (2020). Role of Porcine Aminopeptidase N and Sialic Acids in Porcine Coronavirus Infections in Primary Porcine Enterocytes. Viruses.

[121] Yuan Y.X., Zu S.P., Zhang Y.F., Zhao F.J., Jin X.H., Hu H. (2021). Porcine Deltacoronavirus Utilizes Sialic Acid as an Attachment Receptor and Trypsin Can Influence the Binding Activity. Viruses.

[122] Nova N. (2021). Cross-Species Transmission of Coronaviruses in Humans and Domestic Mammals, What Are the Ecological Mechanisms Driving Transmission, Spillover, and Disease Emergence?. Front. Public Health.

[123] Woo P.C., Huang Y., Lau S.K., Yuen K.Y. (2010). Coronavirus genomics and bioinformatics analysis. Viruses.

[124] Woo P.C., Lau S.K., Huang Y., Yuen K.Y. (2009). Coronavirus diversity, phylogeny and interspecies jumping. Exp. Biol. Med..

[125] Opriessnig T., Huang Y.W. (2020). Coronavirus disease 2019 (COVID-19) outbreak: Could pigs be vectors for human infections?. Xenotransplantation.

[126] Chen W.J., Yan M.H., Yang L., Ding B.L., He B., Wang Y.Z., Liu X.L., Liu C.H., Zhu H., You B. (2005). SARS-associated coronavirus transmitted from human to pig. Emerg. Infect. Dis.

[127] Wang Q., Zhou Z.J., You Z., Wu D.Y., Liu S.J., Zhang W.L., Fan K.R., Luo R., Qiu Y., Ge X.Y. (2021). Epidemiology and evolution of novel deltacoronaviruses in birds in central China. Transbound. Emerg. Dis..

[128] Woo P.C.Y., Lau S.K.P., Lam C.S.F., Lau C.C.Y., Tsang A.K.L., Lau J.H.N., Bai R., Teng J.L.L., Tsang C.C.C., Wang M. (2012). Discovery of Seven Novel Mammalian and Avian Coronaviruses in the Genus Deltacoronavirus Supports Bat Coronaviruses as the Gene Source of Alphacoronavirus and Betacoronavirus and Avian Coronaviruses as the Gene Source of Gammacoronavirus and Deltacoronavirus. J. Virol..

[129] Boley P.A., Alhamo M.A., Lossie G., Yadav K.K., Vasquez-Lee M., Saif L.J., Kenney S.P. (2020). Porcine Deltacoronavirus Infection and Transmission in Poultry, United States. Emerg. Infect. Dis..

[130] Jung K., Hu H., Saif L.J. (2017). Calves are susceptible to infection with the newly emerged porcine deltacoronavirus, but not with the swine enteric alphacoronavirus, porcine epidemic diarrhea virus. Arch. Virol..

[131] Liang Q., Zhang H., Li B., Ding Q., Wang Y., Gao W., Guo D., Wei Z., Hu H. (2019). Susceptibility of Chickens to Porcine Deltacoronavirus Infection. Viruses.

[132] Liu Y., Wang B., Liang Q.Z., Shi F.S., Ji C.M., Yang X.L., Yang Y.L., Qin P., Chen R., Huang Y.W. (2021). Roles of Two Major Domains of the Porcine Deltacoronavirus S1 Subunit in Receptor Binding and Neutralization. J. Virol..

[133] Lednicky J.A., Tagliamonte M.S., White S.K., Elbadry M.A., Alam M.M., Stephenson C.J., Bonny T.S., Loeb J.C., Telisma T., Chavannes S. (2021). Independent infections of porcine deltacoronavirus among Haitian children. Nature.

